# Elastic Critical Resistance of the Simple Beam Grillage Resulting from the Lateral Torsional Buckling Condition: FEM Modelling and Analytical Considerations

**DOI:** 10.3390/ma16041346

**Published:** 2023-02-05

**Authors:** Rafał Piotrowski, Andrzej Szychowski, Josef Vičan

**Affiliations:** 1 Department of Strength of Materials and Building Structures, Faculty of Civil Engineering and Architecture, Kielce University of Technology, 25-314 Kielce, Poland; 2 Department of Structures and Bridges, Faculty of Civil Engineering, University of Zilina, Univerzitna 8215/1, 010 26 Zilina, Slovakia

**Keywords:** elastic critical resistance (ECR), simple beam grillage (SBG), critical beam, critical moment of lateral torsional buckling, elastic restraint at the support joints, FEM simulations

## Abstract

Transversely loaded beam grillages are quite often used in industrial construction. In order to produce a safe design of such structures, it is necessary to account for the lateral torsional buckling phenomenon, which reduces load-bearing capacity. To be able to calculate the relevant reduction factor, the elastic critical load must be determined. As regards the existing design practice for such structures, simplified conditions are assumed for the mutual restraint of the component beams. However, this approach does not correspond to reality. This study discusses the results of numerical investigations and analytical calculations concerning the effect of the elastic action of simple beam grillage (SBG) joints on the critical load, which results from the lateral torsional buckling (LTB) condition. The SBG was defined as a flat system of interconnected beams, unstiffened laterally and loaded perpendicularly to the grillage plane. The analysis covered H-shaped grillages with different span ratios of component beams, in which the main (coupling) beam was decisive for instability. The effectiveness of the use of closed-section stiffeners at the grillage joints was also investigated. The grillage elastic critical resistances (ECR) were determined for two variants of joint stiffening. The computations were performed by means of FEM numerical simulations. The spatial models were discretised with the following elements: (1) solid ones in Abaqus, (2) shell ones in ConSteel, and (3) thin-walled bars in ConSteel. The LTB critical moments of the weakest beam (critical beam), elastically restrained against warping and against lateral rotation (in the LTB plane), were computed using the analytical methods developed by the authors. To this end, the methods were proposed to determine the indexes of the critical beam elastic restraint in the adjacent stiffening beams. In the study, it was demonstrated that (1) taking into account the conditions of mutual elastic restraint and interaction of the component beams provides a more accurate assessment of the grillage ECR, (2) the use of closed-section stiffeners in the grillage joints increase the ECR compared with classic flat stiffeners, (3) the grillage ECR can be estimated based on the critical moment *M_cr_* of the weakest beam (critical beam) when the conditions of its elastic restraint in joints are accounted for.

## 1. Introduction

Methods currently used for the design of steel structures aim to adequately represent the actual performance conditions in the engineering computational models. In particular, this refers to complex structures, in which component members interact in the distribution of loads and redistribution of internal forces. Examples of such structures include steel frames, framework structures, or grillages, especially those with stiff (e.g., welded) joints. When appropriate criteria for the joint properties are met, such structures can be classified as the so-called ‘fully continuous systems’ [1,2].

The present study concerns the elastic critical resistance (ECR) of the H-type simple beam grillage (SBG). The ECR is measured by the external critical load or critical moment (*M_cr_*) from the condition of lateral torsional buckling (LTB) of at least one component beam. The ECR of the grillage constitutes the upper limit of the design resistance and can be used to determine the so-called relative slenderness and the reduction factor for LTB of the weakest (critical) beam. Such a way of determining the beam design resistance, taking into account its imperfections, is permitted in European standards [1]. In order to avoid ambiguity, in this study the H-type SBG was defined as an H-shaped flat system of interconnected beams, which are unstiffened laterally and loaded perpendicularly to the grillage plane. On the other hand, the critical beam was called the grillage weakest beam from the condition of lateral torsional buckling (LTB), which is elastically restrained against warping and against lateral rotation (rotation in the LTB plane) at the grillage joints.

As regards welded grillages, the so-called fully continuous connection solutions, where members of equal or similar heights are connected, make it possible to maintain continuity in the transmission of displacements (including cross-sectional warping) and cross-sectional forces (including bimoments) at the joints. When the computational models of steel grillages account for the effects above, it is possible to design this class of structures in a more effective manner.

The interaction of members connected at the joints of steel frames or framework structures can be taken into consideration in the programs using the finite element method (FEM). They include those based on classic bar elements (with 6 degrees of freedom at each node). In this case, the definitions of the nodal degrees of freedom make it possible to account for the elastic translational and rotational stiffnesses at the structure joints. The warping stiffness, however, is disregarded. For grillages, this approach may prove insufficient, especially for stability analysis. The problem is solved in ConSteel (ConSteel 15) software, where bar thin-walled elements are employed taking into account the additional degree of freedom related to section warping (i.e., 7 degrees of freedom are found at the node). An additional advantage offered by ConSteel is the possibility of converting the grillage bar model into a shell model. This approach allows an even more accurate analysis of the stability loss, although not all properties of the structural details can be fully represented.

In order to directly incorporate the phenomenon of spatial interaction of members (e.g., beams in the grillage structure) while taking into account joint details, it is necessary to develop a spatial FEM model. In the model, shell elements (for cold-formed sections) or solid elements (for hot-rolled sections or their welded equivalents) should be employed, e.g., in Abaqus (Abaqus v. 6.12) software. However, to correctly build an extended model for numerical simulations, which would take the details of solutions of the joint into account, the design engineer must have experience in spatial FEM modelling. The development of such models is generally time-consuming, and additionally significantly extends the time of computations. This way of modelling relatively large parts of the structure, in which joint details are taken into account, significantly increases the size of the task, even for modern computers currently used by engineers.

To optimise numerical models and shorten the time of computations, mixed FEM models are sometimes used. In the models, 1D bar elements of the beams are complemented by 3D joint models. In this respect, static and stability analysis methods reported in studies [3,4,5,6] among others, provide an interesting option. Such solutions allow for the local, spatial modelling of the geometry of joint details, while using a bar model for other structural members. The fundamental benefits brought by those methods is a considerable reduction in the number of finite elements and the duration of computations. At the same time, the elastic restraint of the beams at the joints is still taken into account. However, this does not change the fact that this approach also requires that a designer should have a considerable, and in some cases even greater (in relation to homogeneous models), skill in using the FEM technology.

Another method of spatial modelling of elastic properties of welded corners in flat frames is discussed in [7]. In this case, the connection of I-section bar members of the structure (beam, column) is performed by means of a super element that takes into account the transmission of warping through the joint. In the computational model, the flanges of the joined beam and column and the stiffeners used in the corner of the frame are modelled with simple beam elements. The practical use of the super element, implemented in the ConSteel 15 software, makes it necessary to indicate the joined bar members and to select the stiffening method. The correctness of the solution was verified by FEM using shell elements [8].

Although FEM simulations offer many advantages and possibilities, from the point of view of structural safety, it is reasonable to verify the obtained results by means of performing at least approximate analytical calculations. The solution (e.g., the analysis of spatial grillage stability loss) involves finding the weakest member (the critical beam) that is elastically restrained in the adjacent members (supporting beams). For the critical beam, it is necessary to determine the indexes of the elastic restraint in the stiffening members while the properties of the joints are taken into consideration. The approach is to some extent analogous to that used in the critical plate method (CPM) employed in the analysis of critical buckling resistance and the design of ultimate resistance of thin-walled members susceptible to local instability [9]. The manner and criteria of the division of the beam grillage component elements into critical beams and stiffening beams for more complex arrangements (shapes) are currently being investigated and will be discussed in another study from the same authors.

As regards stability analysis of steel beams, which takes into account different types of elastic restraint at support joints, the solutions proposed, among others, in [10,11,12,13], can be applied. In the stability analysis of the designated beam members of the structure, it is also possible to employ LTBeam (v. 1.0.11) or LTBeamN (v. 1.0.3) software. Like in ConSteel, they use bar finite elements supplemented with the so-called warping degrees of freedom.

The literature on the interaction of component beams in grillages or similar structures (e.g., system of beams braced against torsion) focuses on, among others, (1) lateral torsional buckling of I-girders discretely braced against torsion and/or against translation [14,15,16,17,18,19], including lateral torsional buckling of beams with point bracing [20,21], (2) the numerical analysis of two- and four-beam structural systems transversely braced with coupling beams [22], (3) the assembly of beam systems in skewed bridge structures [23], (4) global stability loss in two-beam systems (beams connected by cross-frames) [24,25], (5) modelling of curvilinear composite bridges, in which the grillage structure made of full-wall I-beam girders was approximated by an equivalent 3D truss model [26], (6) taking into account, in the global analysis, the actual stiffness of the component beam joints (stringer-to-crossbeam) of the deck members of old railway bridges [27]. A considerable number of studies are available in the technical literature that deal with various grillage systems. The component beams of those structures are laterally stiffened by (a) steel diaphragms and timber struts, e.g., [28], (b) a reinforced concrete deck slab (in bridge structures), e.g., [29], (c) sheathing plates in ship deck structures, e.g., [30]. However, studies were not found to provide the analysis of H-type grillages built from beams unstiffened laterally, in which the main beam carrying the transverse load was a coupling beam. Such structures can be seen, among others, in industrial construction as support structures for technological equipment.

The specific objectives of this study are as follows: (1) to evaluate the effect of the span of the component beams, and of the elastic action of the H-type SGB joints on the grillage ECR from the LTB condition, (2) to investigate the effect produced by closed-section stiffeners used at the grillage joints, (3) to examine the possibility of determining ECR in H-type grillage on the basis of elastic stability analysis of the critical beam with the proposed manner of calculation of the elastic restraint indexes while taking into account the properties of the joints. Additionally, one of the goals of this study is to consider the potential ECR reserves of the grillage’s weakest beam. The reserves result from the properties of the joints that are stronger than the fork support. The critical moment (*M_cr_*) of LTB, determined on this basis, can be employed to determine the beam relative slenderness and the reduction factor for LTB in design calculations. For fully continuous beam grillage joints, the adoption of fork support conditions in the computational model can lead to an overly conservative design.

Three approaches (methods) were employed to carry out the task.

Method 1—Numerical simulations in Abaqus using a spatial FEM model for the whole structure (with joint details) with the use of solid (volume) finite elements. In this case, the grillage ECR, for a given load type, was determined from the critical load multiplier obtained from the Buckling procedure.

Method 2—Numerical simulations in ConSteel using a spatial FEM model for the whole structure. The following elements were employed: (a) shell, (b) thin-walled bars (7 degrees of freedom at a node). In this case, the grillage ECR, for a given load type, was obtained from the elastic lateral torsional buckling analysis.

Method 3—Designation of the critical beam from the grillage structure. The beam is decisive for stability loss of the grillage. For the critical beam, the elastic restraint conditions in the adjacent (stiffening) beams were taken into consideration. The grillage ECR was determined on the basis of critical moment *M_cr_* of lateral torsional buckling of the critical beam. After the indexes of the beam elastic restraint in the support joints were determined, *M_cr_* was found. This was carried out using three methods, namely (1) analytical calculations, in which algorithms based on the energy method were used, (2) approximation formulas, and (3) LTBeamN (FEM) software.

It is important to show the congruence between advanced FEM simulations for the whole structure and analytical calculations for a designated critical beam. When computational methods are doubled, it is possible, e.g., to identify potential errors in the structure modelling. The possibility of using relatively simple approximation formulas allows a more advanced preliminary design. For basic structural systems, the formulas can also be employed in the standard design. This approach improves the safety of structures already at the design stage.

The study provides a detailed analysis of H-type SBG for strictly determined parameters. They account for the following: (1) the grillage geometry and diagram, (2) the restraint method and four span variants of the support beams, (3) two span alternatives of the main (coupling) beam, (4) type of the cross-section of the component beams, (5) the method of joint stiffening, (6) the load type and ordinates of the load application point. It is obvious that the grillage ECR is a function of the assumed variables, which translates into the complexity of the problem. A multi-criteria analysis of different grillage types, including a wider range of adopted parameters (variables), will be discussed in the authors’ publications in the future.

This study is an introduction to the ‘critical beam’ method. The designated beam is selected from the structural system on the basis of component stability analysis.

The innovativeness of the research reported in this study, in relation to solutions known from the literature, involves the following:

(i) A departure from fork support, classically adopted in beam stability analysis;
(ii) Taking into account the interaction of component beams and joint properties (including the stiffening type) in the ECR analysis of H-type grillage;
(iii) Proposing a method for the determination of the indexes of the critical beam elastic restraint in stiffening beams;
(iv) Demonstrating that the ECR of H-type grillage can be determined based on the critical moment of the critical beam. Further, on the basis of *M_cr_*, it is possible to determine the so-called relative slenderness and reduction factor for LTB (taking into account the equivalent imperfection according to [1]) of the design resistance of the beam against LTB;
(v) Demonstrating that the approach based on the critical beam analysis offers a more accurate representation of the SBG’s actual performance in the elastic LTB phase while using a relatively simple analytical model.

## 2. Diagram of the Analysed Structure

**Figure 1:**
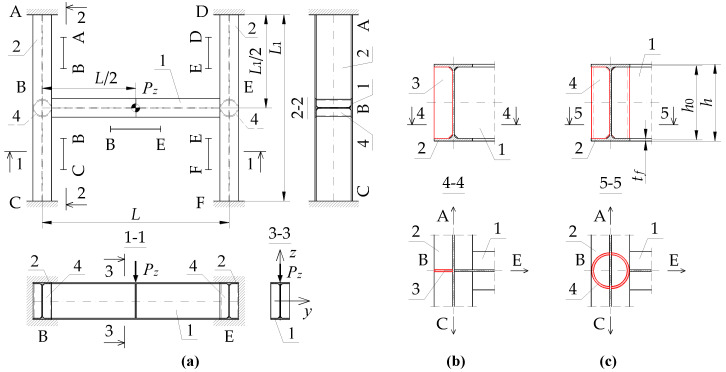
Diagram of the grillage: (**a**) view from the top, (**b**) flat stiffener (*t_p_* = 8 mm), (**c**) closed-section stiffener (Ø139.7/8). Notation: 1—main (coupling) beam, 2—support beam, 3—flat stiffener, 4—closed-section stiffener [33].

The detailed analysis concerned an SBG of H-type, composed of support beams A–C and D–F, and the main (coupling) beam B–E (Figure 1a). The component beams of the grillage were produced from an IPE300 (*E* = 210 GPa, *ν* = 0.3, *G* ≅ 81 GPa) section for two main beam span variants, namely *L* = {6, 7.5} [m], and four variants of support beam spans *L*_1_ = {3, 6, 9, 12} [m]. For each of the span variants, two types of stiffening were taken into consideration at joints B and E, namely flat stiffeners (Figure 1b) and closed-section stiffeners (Figure 1c). These two methods of stiffening are characterised by a large disparity in the obtained warping stiffness [31,32]. It was assumed that the homogeneous grillage beams were weld-connected at joints B and E. In this way, it was possible to obtain full continuity in the transmission of displacements, including warping, and in the transmission of cross-sectional forces, including bimoments. It was assumed that the resistance of the welded connections is not lower than that of the connected beams. At this stage of the analysis, the effect of post-weld stresses and deformations in members under bending was disregarded.

Loading diagrams frequently found in engineering practice for beam B–E were analysed: (a) force concentrated at midspan, (b) uniformly distributed load, (c) load distributed over a triangle. Unit loads were applied to the top of the upper flange (*z_g_* = +*h*/2) of the beam. In addition, beams A–C and D–F were loaded with reactions from the beam B–E. Beams A–C and D–F were assumed to be fully fixed on supports A, C, D and F. Based on static calculations performed with Autodesk Robot Structural Analysis Professional and ConSteel, it was found that the distribution of the moment *M_y_* on the beam B–E corresponds approximately to the free support. Additionally, it does not indirectly affect the critical moment of the lateral–torsional buckling of the B–E beam. This results from the low torsional stiffness of the open-section support beams (A–C, D–F) for the spans considered in the analysis.

In this case, the transversely loaded main (coupling) beam is decisive for the ECR of the H-grillage from the lateral torsional buckling condition. This stems from a comparison of the ECR of the component beams selected from the structure. For the grillage of concern, the main beam critical moment is at least a few times smaller than the *M_cr_* of the support beams completely fixed at the support joints and coupled with the B–E beam. Therefore, the main (coupling) beam of the analysed grillage (Figure 1) is its critical beam. The method behind this approach is suitable for more complex grillage structures, and it involves designating the ‘critical beam’ which is elastically restrained in the adjacent stiffening beams. The method will be discussed in detail in another paper.

## 3. FEM Simulations in Abaqus Software (Method 1)

**Figure 2:**
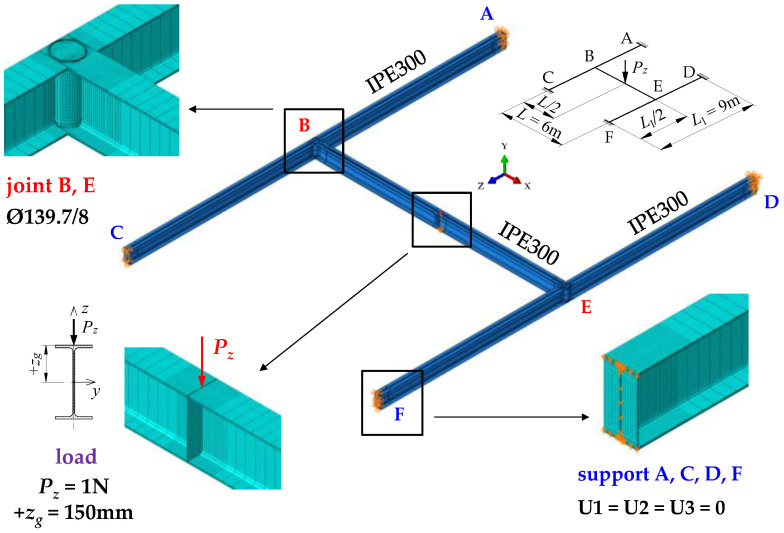
The spatial model of exemplary H-grillage obtained with Abaqus.

FEM simulations in Abaqus (Abaqus v. 6.12) [34] involved the grillage modelled as a whole, including physical representation of the joint details. To discretise the beam members and joints, volumetric finite elements of the C3D8R type were used. They had eight nodes with six degrees of freedom at a node. The beams were discretised using a basic FEM mesh with an average mesh size of 50 × 10 × *t*/4 [mm] (where *t* is wall thickness). The mesh was compacted at the structure problematic points to 10 × 10 × *t*/4 [mm]. Finite element compaction occurred at supports (A, C, D, and F), at stiffened intermediate joints (B and E), and at the point where concentrated load was applied. The number of volumetric finite elements of the C3D8R type depends on the span of the grillage component beams (A–C, B–E, D–F) and the method of stiffening at joints B and E. For the grillage shown in Figure 2, the Abaqus software generated 192,748 finite elements. The boundary conditions of the support beams took into account the prevention of translation, rotation and warping of the support sections with respect to the principal axes of inertia. This effect was achieved through translation blockage in three directions (U1 = U2 = U3 = 0) imposed on the frontal planes of the support cross-sections of the beams.

The transverse load was applied to the top of the upper flange of the main beam B–E. The load had the following form: (a) a concentrated force, modelled as a concentrated load applied to one mesh node, (b) a uniformly distributed load, modelled as a concentrated load (in the *z*-axis) of constant value, distributed along the entire length of the beam, (c) a load distributed over a triangle, modelled as a concentrated load (in the *z*-axis), the value of which varied linearly. The way of modelling different parts of the structure of the exemplary H-grillage is shown in Figure 2. The computations were performed for the elastic range, using a computational step of the Buckling procedure.

## 4. FEM Simulations in ConSteel Software (Method 2)

ConSteel (ConSteel 15) software [35] was employed to model the grillage in a 3D spatial system. A bar model and a shell model based on it were used.

### 4.1. The Bar Model

For the spatial bar model of the H-grillage, the component beams were modelled using modified thin-walled elements (7 DOF) that accounted for the section warping at the nodes (bar elements with 7 degrees of freedom at a node). In ConSteel, the warping function of the thin-walled section is based on the Vlasov theory of thin-walled beams [36].

**Figure 3:**
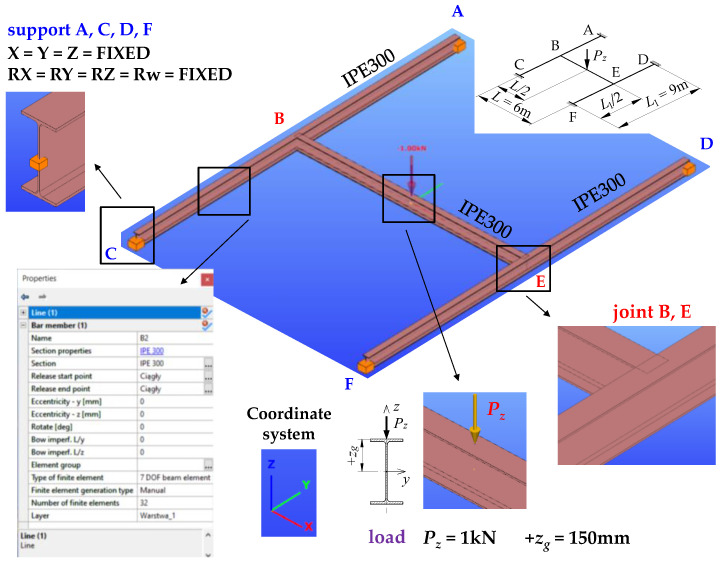
The bar spatial model of exemplary H-grillage obtained with ConSteel.

Sections of all the grillage beams were represented by the IPE300 profile, which was retrieved from the program library of sections. Each of the beams (A–C, D–F, and B–E) were discretised with 32 finite elements. A certain limitation on the use of hot-rolled sections (when modelling the structure with thin-walled elements 7 DOF) lies in the fact that it is not possible to define stiffening at joints B and E and under concentrated force. Consequently, the grillage ECR was estimated without taking into account the warping stiffness of the stiffeners (Figure 3).

As regards the modified thin-walled element 7 DOF, flat stiffeners or closed-section stiffeners (2C, 2L) can only be implemented in numerical models of welded I or H members. For the warping stiffness of such a stiffener to be included in the computations of the beam elastic critical resistance, the stiffener must be defined on both sides of the web. If a flat or closed-section stiffener is used only on one side of the web, its effect is disregarded by the program.

The effect of stiffeners in the joints of the H-shaped grillage modelled with bar elements (7 DOF), where hot-rolled sections are used, can be taken into account indirectly. One of the methods consists of a local (only within a joint) modification of the cross-section’s geometry of mutually transverse elements converging in a joint. In such a case, the length of the equivalent beam element and the necessary geometrical characteristics of the modified cross-section should be determined. In this study, when determining the ECR of the grillage, the hot-rolled cross-sections retrieved from the program library were not modified.

The transverse load was applied to the top of the upper flange of the B–E main beam, in the z-z axis of the beam. The load was of the following form: (a) a point load applied at the midspan, (b) a load uniformly distributed along the length, (c) a load nonuniformly distributed (over a triangle) along the length. The method of modelling the structure of exemplary H-grillage is shown in Figure 3. The computations were performed in the elastic range, using the buckling analysis.

### 4.2. The Shell Model

The function converting section walls of a bar element into a shell was implemented in the ConSteel (ConSteel 15) [35] software. Owing to that, the grillage spatial model was also developed. The model employed triangular shell finite elements with 6 degrees of freedom at a node. While generating the shell model, the grillages originally modelled with thin-walled 7 DOF elements (Section 4.1) were used. This way of generating shell element mesh is extremely efficient. However, as it will be shown later, some inaccuracies occur in the conversion of the geometric characteristics of the hot-rolled open section to the thin-walled shell section.

In the conversion of the IPE300 section to the shell model, the web area with root radius was replaced with an equivalent shell element of greater thickness. After the conversion of the full grillage model to the shell model, it was necessary to cut the flanges of the main beam (B–E) so that it would fit the flanges of the support beams (A–C, D–F).

The subsequent stage involved the modelling of the stiffeners as flat or closed shells at joints B and E. That made it possible to explicitly take into account the warping stiffness of the stiffeners when determining the ECR of the grillage. In the conversion to the shell model, the boundary conditions of the bar model support beams, which took into consideration full fixity, i.e., blockage of all 7 degrees of freedom, were automatically transformed into the appropriate support constraints of the shell model. At the support points of the support beams, over their cross-section contour, the so-called rigid bodies were generated. They connected the cross-section contour to the support point, at which full fixity conditions were imposed.

At the final stage, the program discretised all the grillage members (beams, stiffeners) using three-node/triangular finite elements, with six degrees of freedom at each node. The average mesh dimension of 20 mm was imposed. The mesh was automatically compacted at the problematic points of the structure.

**Figure 4:**
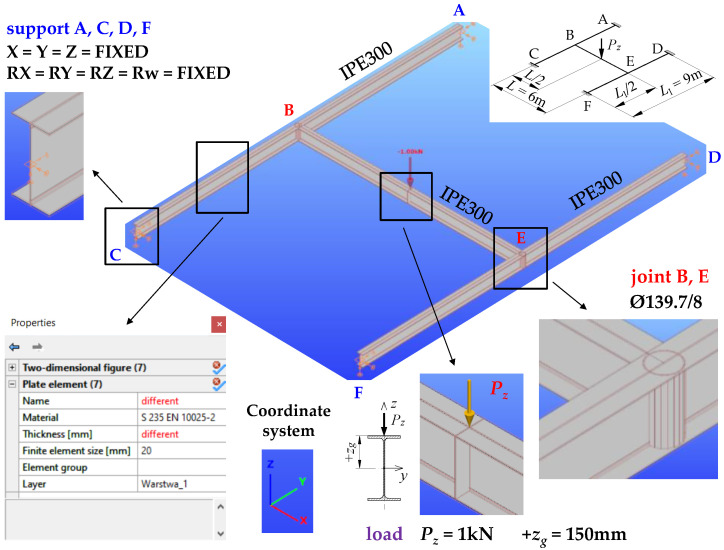
The spatial model of exemplary H-grillage obtained with ConSteel after the conversion to shell elements.

The manner of load application was the same as in the bar model (Section 4.1). The shell model of the structure of exemplary H-grillage is shown in Figure 4. The computations were performed in the elastic range, using the buckling analysis.

## 5. The Model of Designated Critical Beam (Method 3)

### 5.1. Conditions of Elastic Restraint

In order to account for the spatial interaction of beam members of the SBG of H-type (Figure 1), the parameters of the elastic restraint of the main (critical) beam nodes in the support (stiffening) beams were determined.

Elastic restraint against warping and elastic restraint against lateral rotation of the B–E critical beam at the grillage joints (B and E) were taken into account by introducing dimensionless indexes of restraint [11]:

(a) against warping:

(1) $$\kappa_{\omega }=\frac{\alpha_{\omega }L}{2EI_{\omega }+\alpha_{\omega }L}\;\;,\;$$

and (b) against lateral rotation:

(2) $$\kappa_{u}=\frac{\alpha_{u}L}{2EI_{z}+\alpha_{u}L}\;\;,$$

where *α_ω_*—stiffness of the elastic restraint against warping acc. [11], *α_u_*—stiffness of the elastic restraint against lateral rotation acc. [11], *L*—beam span, *I_ω_*—warping constant, *I_z_*—second moment of area about the minor axis (*z*-axis), *E*—Young’s modulus.

The restraint index *κ_ω_* (1) ranges from *κ_ω_* = 0 for full warping freedom to *κ_ω_* = 1 for complete warping blockage. Additionally, the restraint index *κ_u_* (2) varies from *κ_u_* = 0 for full freedom of lateral rotation to *κ_u_* = 1 for complete lateral rotation blockage.

**Figure 5:**
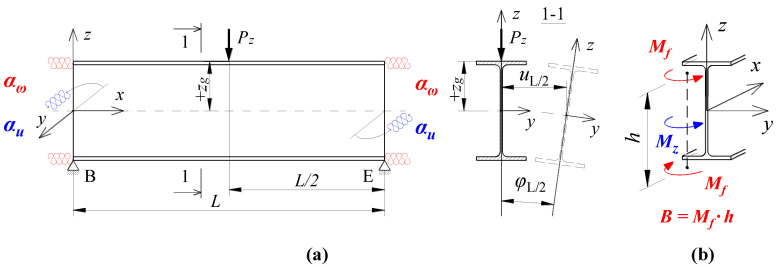
The designated critical beam B–E of the SBG of H-type: (**a**) static diagram and boundary conditions, (**b**) bimoment *B* and moment *M_z_* at the support nodes [13].

For the SBG of concern (Figure 1a), with beams A–C and D–F restrained at the supports, the elastic restraint against warping (*α_ω_*) and the elastic restraint against the lateral rotation (*α_u_*) of the critical beam at joints B and E, resulting from the elastic actions of the support beams (Figure 5), can be written with the following formulas:

(3) $$\alpha_{\omega }=\overset{2}{\underset{i=1}{\sum }}\frac{4EI_{\omega }^{i}}{L_{i}}\;\;,\;$$

(4) $$\alpha_{u}=\overset{2}{\underset{i=1}{\sum }}\frac{4EI_{z}^{i}}{L_{i}}\;\;,$$

where *I_ω_^i^*, *I_z_^i^*,—geometric characteristics of the *i*-th sections of the supporting beams A–B, B–C, D–E, and E–F, reaching the joints B and E, *L_i_*—the span of the A–B and B–C compartments of the A–C beam and D–E and E–F compartments of the D–F beam.

Formulas (3) and (4) were written in a manner analogous to that used in the classic Cross method [37].

In addition to the advantageous effect of adequate stiffnesses of the beams in contact (Figure 1a), the stiffness of the grillage critical beam elastic restraint against warping (*α_ω_*) at joints B and E is also affected by the way the joints are stiffened (Figure 1b,c). The additional stiffness *α_ω_^R^*, related to the selected geometry of the joint stiffening, was given by the following formula [32]:

(5) $$\alpha_{\omega }^{R}=GI_{d}h_{0}\;\;,\;$$

where *G*—Kirchhoff’s modulus, *I_d_*—torsion constant of stiffener (Saint-Venant torsion constant), *h*_0_ = *h* − *t_f_*—theoretical stiffener height between flange axes (Figure 1c).

The total elastic restraint against warping *α_ω_* of the support sections of the critical beam B–E is expressed as follows:

(6) $$\alpha_{\omega }=\overset{2}{\underset{i=1}{\sum }}\frac{4EI_{\omega }^{i}}{L_{i}}+\alpha_{\omega }^{R}\;\;.\;$$

Two different types of stiffening were employed in the grillage welded joints B and E (Figure 1).

The analysis of the warping stiffness of different types of stiffeners [31,32] showed that the effect produced by curbing the beam section’s warping was negligibly small when flat stiffeners (of commonly found thicknesses) were used, compared with closed-section stiffeners. Consequently, the contribution of flat stiffeners *t_p_* = 8 mm, located in the axis of the B–E critical beam (Figure 1b), was neglected when the stiffness of the beam elastic restraint against warping *α_ω_* (6) was determined, i.e., it was assumed that *α_ω_^R^* = 0. Conversely, closed-section stiffeners (Figure 1c), especially those made from tubes with a diameter corresponding to the width of the beam flanges (and of the same thickness as the flat stiffeners), are highly effective in reducing the warping of both the beam cross-section [31] and the grillage welded joint. It is possible to treat closed-section stiffeners as additional details of the joint structure, the properties of which can be included in the analytical model. Such a model could effectively complement FEM models based on bar elements.

With respect to grillages with similar strength utilisation in component beams (the critical beam and stiffening beams), a reduction in the appropriate stiffnesses of the support beams (stiffening the critical beam), including the closed-section stiffeners, may be important and should be taken into account. Dealing with such cases will be discussed in detail in further studies by the authors.

At the current stage of investigations into the problem, the reduction in stiffness against warping and against the lateral rotation of the restraining beams, including closed-section stiffeners, can be conservatively estimated following the formulas below:

(7) $$\alpha_{\omega }{}^{*}=\alpha_{\omega }\left(1-\frac{M_{cr}}{M_{cr,u}}\right)\;\;,\;$$

(8) $$\alpha_{u}{}^{*}=\alpha_{u}\left(1-\frac{M_{cr}}{M_{cr,u}}\right)\;\;,$$

where *M_cr_*—critical moment (critical load) from the condition of the critical beam lateral torsional buckling, *M_cr,u_*—critical moment (critical load) from the condition of the lateral torsional buckling of the supporting beams.

The computational example (Appendix A) shows how the concept could be applied to the design practice.

### 5.2. The Critical Moment of the Lateral Torsional Buckling of the H-Grillage Main Beam

Critical moments of the lateral torsional buckling *M_cr_* of the critical beam B–E, elastically restrained against warping and against lateral rotation (in the lateral torsional buckling plane) at fixed support beams A–C and D–F (Figure 1a), were determined using the M_LTB,EL,2_ algorithm. It was developed by the authors and written in Mathematica software. To determine the critical moments, the approximation formula derived in [11] was also used. The ECR of the critical beam B–E was also determined using the LTBeamN software.

#### 5.2.1. The Analytical Model—The Energy Method

The energy method in variation terms was used to derive the analytical formulas of the M_LTB,EL,2_ algorithm. The total potential energy of the beam-load system was minimisation using the Rayleigh–Ritz method. The torsion angle function (*φ*) and the beam lateral deflection function (*u*) were approximated by originally coupled (using the fixity indexes *κ_ω_* and *κ_u_*) power polynomials with simple static interpretation. The first three terms of the polynomial series were employed, within which polynomials describing the deflection of the simply supported beam were coupled with polynomials describing the deflection of the bilaterally restrained beam [11]. The formulas of the functions *φ*(*κ_ω_*) and *u*(*κ_u_*) made it possible to independently consider the elastic restraint against warping (0 ≤ *κ_ω_* ≤ 1) and the elastic restraint against lateral rotation (0 ≤ *κ_u_* ≤ 1).

#### 5.2.2. Analytical Model—Approximation Formula

The critical moment *M_cr_* of the LTB of the beam, elastically restrained against warping and lateral rotation (in the lateral torsional buckling plane), can also be determined based on the approximation formula derived in [11]. The formula draws on symbolic calculations carried out in the M_LTB,EL,2_ algorithm. In this case, only the first coupled term of the polynomial series was used to approximate the torsion angle function *φ*(*κ_ω_*) and the lateral deflection function *u*(*κ_u_*). Still, a good congruence between the *M_cr_* results obtained in [11] and those produced by FEM (LTBeamN) was found.

The approximation formula was written as follows [11]:

(9) $$M_{cr}=D_{1}\frac{-B_{1}EI_{z}z_{g}+\sqrt{EI_{z}\left(B_{2}GI_{t}L^{2}+B_{3}EI_{\omega }+B_{1}^{2}EI_{z}z_{g}^{2}\right)}}{B_{4}L^{2}}\;\;,\;$$

where *B*_1_, *B*_2_, *B*_3_, *B*_4_, and *D*_1_—coefficients depending on the beam loading diagram and the indexes of the elastic restraint *κ_ω_* (1) and *κ_u_* (2) acc. [11], *z_g_*—ordinate of the transverse load application point (Figure 5), *I_t_*—Saint-Venant torsional constant; the remaining symbols were explained earlier in the study.

#### 5.2.3. Thin-Walled Bar FEM Model—LTBeam, LTBemN

The critical moment of the LTB of the grillage critical beam B–E (Figure 1a) can be also determined using the LTBeam [38] and LTBeamN software tools. They are based on thin-walled bar elements (FEM).

LTBeam and LTBeamN are widely known engineering programmes used for determining critical moments of the LTB of beams with arbitrary mono- or bisymmetric I-sections. They make it possible to assume classical boundary conditions (fork support), and also to account for various types of beam stiffening at the support and for complex loading diagrams. In the program default settings, the analysed beam is discretised using 100 finite bar (thin-walled) elements.

In the case under consideration, the interaction of the critical beam B–E with the support beams A–C and D–F should be taken into account by inputting the data on stiffness coefficients of the elastic restraint against warping *α_ω_* (6) and against lateral rotation *α_u_* (4).

**Figure 6:**
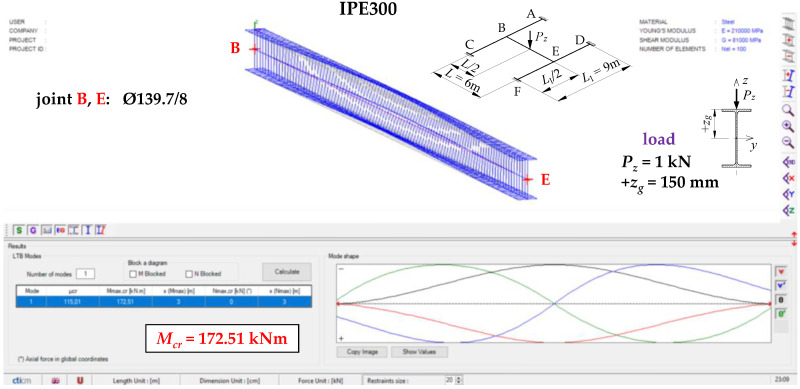
The ECR and the mode of lateral torsional buckling of the critical beam B–E of H-grillage (IPE300, *L* = 6 m, *L*_1_ = 9 m) acc. LTBeamN.

The results of computations in LTBeamN for the critical beam of the exemplary H-grillage (IPE300, *L* = 6 m, *L*_1_ = 9 m, *α_ω_* = 384.41 kNm^3^/rad, *α_u_* = 2254.9 kNm) are shown in Figure 6.

## 6. Selected Results of Computations

### 6.1. Method 1—Volumetric Model—Abaqus

**Figure 7:**
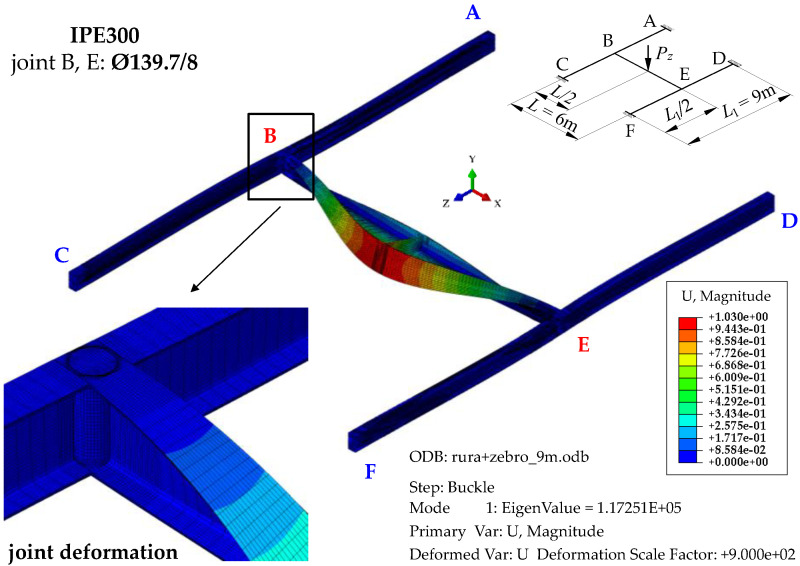
The mode of stability loss in exemplary H-grillage (IPE300, *L* = 6 m, *L*_1_ = 9 m) with closed-section stiffeners at joints B and E, modelled in Abaqus.

Figure 7 shows the mode of stability loss in an exemplary SBG of H-type (IPE300, *L* = 6 m, *L*_1_ = 9 m) with closed-section stiffeners at joints B and E (Figure 1c). The stability loss mode is associated with the first eigenvalue of the ECR measured by the external critical load *P_z,cr_*. It was obtained through Abaqus.

The mode of H-grillage stability loss (Figure 7) acc. the first eigenvalue *P_z,cr,_*_1_ = 117.3 kN shows considerable flexural–torsional deformations (lateral torsional buckling) of the main beam B–E, having the shape of one half-wavelength. This confirms the adopted assumption that concerned the designation (isolation) of the critical beam. Based on further FEM simulations, it was found that the spatial (flexural–torsional) stability loss in the support beams corresponds to the fourth eigenvalue that features the critical load of *P_z,cr,_*_4_ = 1170.2 kN. The second and third eigenvalues of the critical load correspond to the lateral torsional buckling of the main (critical) beam B–E acc. two (*P_z,cr,_*_2_ = 415.6 kN) and three (*P_z,cr,_*_3_ = 823.2 kN) buckling half-wavelengths, respectively.

Table 1 lists exemplary results of ECR computations of the H-grillage for uniformly distributed loads (*q_z,cr_*), which were obtained with Abaqus.

**Figure 8:**
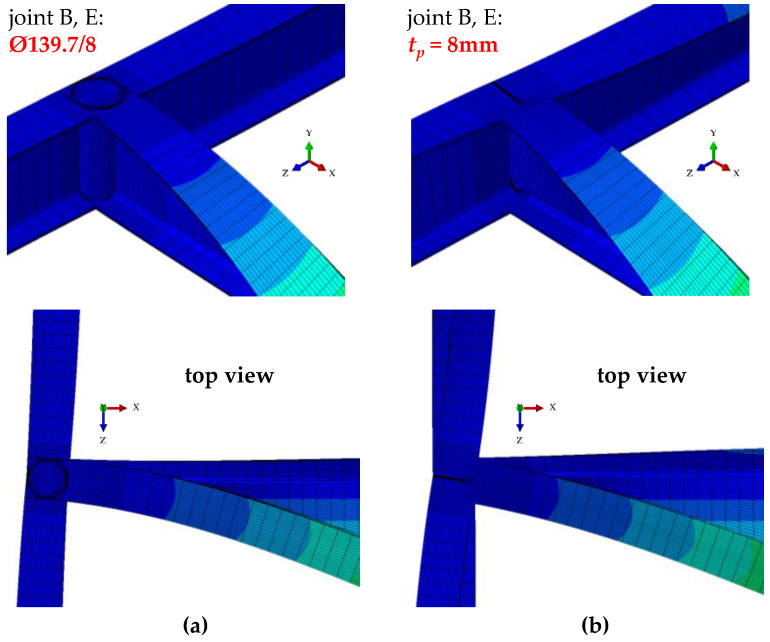
Bimoment deformations of B joint of H-grillage obtained with Abaqus: (**a**) closed-section stiffener Ø139.7/8, (**b**) flat stiffener *t_p_* = 8 mm.

Figure 8 shows joint B deformation (acc. Figure 7) for the stiffening variants analysed in the study (Figure 1b,c). Compared with a flat stiffener (Figure 8b), the use of a closed-section stiffener (Figure 8a) significantly reduced the warping of the near-node cross-section of the B–E main (critical) beam and reduced bimoment transmission (and warping) to the near-node cross-sections of the support beams (A–C, D–F). This is related to the considerable torsional rigidity of the closed-section stiffener Ø139.7/8. The stiffener is loaded in the planes of the IPE300 beam flanges with opposite moments *M_f_*, generated by bimoment *B* (see Figure 5b). In this case, closed-section stiffeners (Figure 8a) act as local bimoment elastic support. By contrast, the poor torsional rigidity of the flat stiffeners, loaded with moments *M_f_*, results in lower ECR values of the H-type grillage *q_z,cr_* (see Table 1) and much greater lateral displacements and torsion of the support beams (Figure 8b).

### 6.2. Method 2—ConSteel

#### 6.2.1. The Bar Model (Thin-Walled Bar Elements)

**Figure 9:**
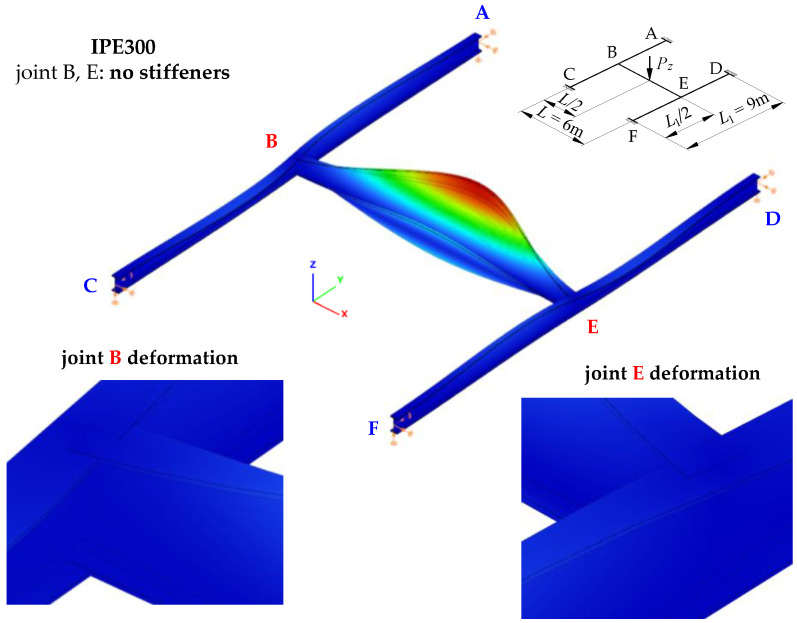
The mode of stability loss in exemplary H-grillage modelled in ConSteel software—thin-walled bar model.

Figure 9 shows the mode of stability loss of an exemplary SBG of H-type associated with the first eigenvalue of the critical resistance according to the ConSteel software (version 15). (It should be noted that because of the homogeneous hot-rolled profiles (IPE300) used in the structure (Section 4.1), stiffeners in joints B and E of the analysed grillages in ConSteel were not included.)

As in the case of Figure 7 (analysis according to Abaqus software), the mode of the H-grillage stability loss (Figure 9) according to the first eigenvalue determined by the ConSteel program (*P_z,cr,_*_1_ = 103.6 kN) is characterized by significant lateral torsional deformations of the B–E main beam. This confirms the adopted assumption concerning the identification (designation) of the so-called the critical beam. Further FEM simulations, this time in the ConSteel software, demonstrated that the spatial (lateral–torsional) loss of stability of the supporting beams also corresponds to the fourth eigenvalue related to the critical load of *P_z,cr,_*_4_ = 1027.2 kN. In this case, (similarly to the simulations according to the Abaqus software), the second and third eigenvalues of the critical load correspond to the lateral torsional buckling of the B–E main (critical) beam according to two (*P_z,cr,_*_2_ = 381.3 kN) or three (*P_z,cr,_*_3_ = 738.0 kN) of half-wavelengths of the lateral buckling.

Table 2 lists the results of calculation of the ECR of the H-grillage for a uniformly distributed load (*q_z,cr_*). The results were determined using ConSteel software—bar model.

#### 6.2.2. The Shell Model

**Figure 10:**
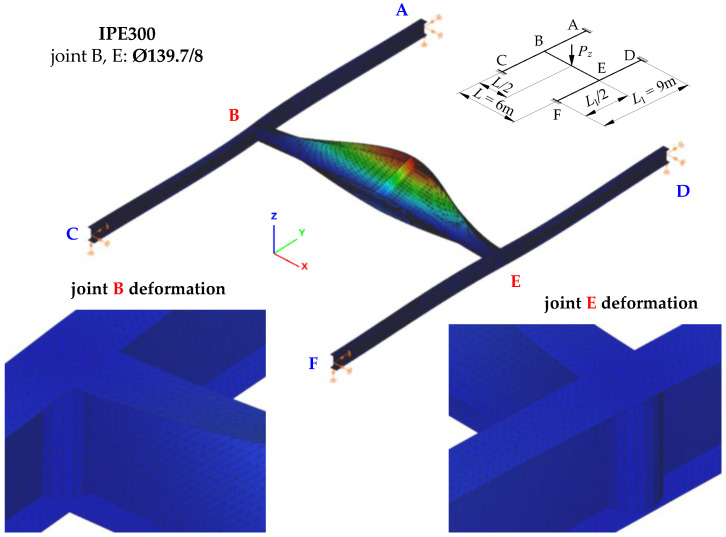
The mode of stability loss in exemplary H-grillage modelled in ConSteel software—shell model.

Figure 10 shows the mode of the stability loss of an exemplary SBG of H-type associated with the first eigenvalue of the ECR according to the ConSteel software—shell model. In this case, the stiffeners (flat or closed) at joints B and E were ‘physically’ modelled as shell elements.

Similarly to the cases illustrated in Figure 7 and Figure 9, the mode of the H-grillage stability loss (Figure 10) according to the first eigenvalue (*P_z,cr,_*_1_ = 110 kN), determined by the ConSteel software, the shell model is characterized by significant lateral torsional deformations of the main beam B–E. As in previous cases, this confirms the adopted assumption about the identification of the so-called critical beam. Based on further FEM simulations (ConSteel—shell model), it was found that the spatial (lateral–torsional) loss of stability of the supporting beams corresponds in this case to the 10th eigenvalue, which is related to the critical load of *P_z,cr,_*_10_ = 1152.9 kN. This results from the fact that in the shell model, the eigenvalues from four to nine refer to various modes of local stability loss of the B–E beam, which were not taken into account in the bar model. As it is not possible to model the roundings in the flange–web connection (in the shell model), the susceptibility of the element to local stability loss increases considerably.

Additionally, the second (*P_z,cr,_*_2_ = 400.3 kN) and the third (*P_z,cr,_*_3_ = 795.6 kN) eigenvalues of the critical load, similarly to the two previous simulations, correspond to the lateral torsional buckling mode of the B–E main (critical) beam according to two or three half-wavelength, respectively.

Table 3 shows the results of calculations of the ECR of the H-grillage for a uniformly distributed load (*q_z,cr_*). The results were obtained with the ConSteel software—shell model.

### 6.3. Method 3—Designated Critical Beam

Determining the ECR of the grillage on the basis of the elastic critical resistance of the weakest beam involves the designation of the critical beam and taking into account the conditions of the beam elastic restraint in stronger stiffening beams. As a part of this method, the ECR of the critical beam was determined based on analytical models and the FEM thin-walled bar model (LTBeamN).

#### 6.3.1. Analytical Models

As a part of the analytical models (Section 5.2.1 and Section 5.2.2), the critical moment *M_cr_* of lateral torsional buckling of the critical beam B–E was determined using the M_LTB,EL,2_ algorithm and the Formula (9) proposed in [11]. The degree of elastic restraint against the warping and lateral rotation of the critical beam at joints B and E was accounted for by means of dimensionless restraint indexes *κ_ω_* (1) and *κ_u_* (2).

Examples of the calculation results of the critical moment *M_cr_* of the uniformly loaded critical beam B–E are given in Table 4.

#### 6.3.2. The Bar Model (FEM)—Thin-Walled Elements (LTBeamN)

As part of the FEM thin-walled bar model (Section 5.2.3), the LTBeamN software was used to determine the critical moment *M_cr_* of the critical beam B–E of the H-grillage. The conditions of elastic restraint of the critical beam in joints B and E were taken into account by the stiffness coefficients: (1) against warping *α_ω_* according to Formula (6), and (2) against lateral rotation *α_u_* according to Formula (4).

Examples of the calculation results of the critical moment of lateral torsional buckling of the grillage critical beam, loaded uniformly, are given in Table 5.

## 7. Comparative Analysis

Table 6 compares the ECR of the H-type grillage determined using Method 1 and Method 2. The grillage main (critical) beam was loaded with a concentrated force at the midspan. The results were obtained from FEM numerical simulations for 3D spatial models. The results from the solid model (Method 1—Abaqus) were taken as reference. The solid finite elements made it possible to model all important details of the spatial structure of the grillage. The solid model took into account both the details of the rounding of the flange–web connection, and also the details of the stiffening stiffeners.

The comparison of the values in Table 6 shows that the use of closed-section stiffeners results in an increase in the ECR of H-grillages from 3.9% (*L* = 7.5 m, *L*_1_ = 3 m) to 15.2% (*L* = 6 m, *L*_1_ = 12 m) according to Abaqus (Column VI), and from 2.9% (*L* = 7.5 m, *L*_1_ = 3 m) to 17.6% (*L* = 6 m, *L*_1_ = 12 m) according to ConSteel—shell model (Column VIII).

With respect to flat stiffeners (*t_p_* = 8 mm), the ECR of the grillages (Table 6) determined for the bar model (Column VII)—ConSteel software (Method 2) gave a very good approximation of the *P_z,cr_* determined for the solid model (Column VI)—Abaqus software (Method 1). The maximum differences did not exceed −0.9%. By contrast, the results from the ConSteel software—shell model (Column VIII)—are lower from −6.1% (*L* = 6 m, *L*_1_ = 3 m) to −9.6% (*L* = 7.5 m, *L*_1_ = 12 m). This resulted from some shortcomings of converting the hot-rolled section bar model to the shell model. The procedure of selecting the equivalent shell thickness within the rounding does not fully transfer the torsional stiffness characteristics of the hot-rolled section to its shell model. Similar differences are also found in the calculation of the critical moment, e.g., of simply supported beams modelled in ConSteel with bar elements (7 DOF) and shell elements using the classic conversion procedure.

For closed-section stiffeners (Ø139.7/8), the results obtained from the thin-walled bar model in ConSteel (Column VII) are lower from −4.2% (*L* = 7.5 m, *L*_1_ = 3 m) to −13.5% (*L* = 6 m, *L*_1_ = 12 m) compared with the results obtained from Abaqus model. As already mentioned (Section 4.1 and Section 6.2.1), results from the fact that the calculations using the bar model did not take into account the closed-section stiffeners at joints B and E. However, the use of the shell model (ConSteel, Column VIII), which allows ‘physical’ modelling of closed-section stiffeners, improves the results of fitting to the Abaqus solid model, with differences ranging from −5.8% (*L* = 6 m, *L*_1_ = 6 m) to −9.5% (*L* = 7.5 m, *L*_1_ = 3 m).

In order to compare the results obtained with Method 1 and Method 3, the critical loads of the grillages obtained from the FEM numerical simulations according to Abaqus, were converted into the equivalent critical moments *M_cr,Abq_* of lateral torsional buckling of the critical (main) beam. The lateral torsional buckling of the B–E critical beam determines the ECR of the grillage. Such conversion is also useful when checking the design resistance of the beam with the reduction factor method (*M_cr_* is needed to determine the beam relative slenderness, on the basis of which the value of the reduction factor for lateral torsional buckling is calculated).

As already mentioned, due to the slight torsional stiffness of the supporting beams A–C and D–F in relation to the bending stiffness of the grillage main (critical) beam B–E, the support moments of the critical beam at joints B and E are insignificant (i.e., they did not exceed 3% of the maximum value of the midspan moment) and may be conservatively disregarded in the design practice. Consequently, the following formulas can be used to convert the critical loads (*P_z,cr_*, *q_z,cr_*, *q_z,t,cr_*) of grillages into the critical moment (*M_cr_*) of lateral torsional buckling of the critical beam B–E: *M_cr_* = (1/4)·*P_z,cr_*·*L*, *M_cr_* = (1/8)·*q_z,cr_*·*L*^2^ and *M_cr_* = (8/125)·*q_z,t,cr_*·*L*^2^.

Table 7 compares the critical moments *M_cr_* of lateral torsional buckling of the H-grillage B–E critical beam, which is uniformly loaded at the level of the top flange (*z_g_* = +*h*/2). The values of the moment were determined for two variants of stiffening of joints B and E (Figure 1b,c) according to (a) Abaqus software (Column VI), (b) LTBeamN software (Column VII), (c) the M_LTB,EL,2_ algorithm (Column IX) and (d) approximation Formula (9) [11] (Column XII). The calculation results obtained from the Abaqus software were taken as reference.

Critical moments (Table 7) determined with the M_LTB,EL,2_ algorithm and Formula (9) [11] gave a very good approximation of the results obtained from FEM software (Abaqus, LTBeamN). The maximum differences were, respectively, M_LTB,EL,2_ vs. Abaqus from −3.0% to +1.8%, Column X, M_LTB,EL,2_ vs. LTBeamN from +0.1% to +0.3%, Column XI, Formula (9) vs. Abaqus from −3.8% to +0.6% (Column XIII), Formula (9) vs. LTBeamN from −1.3% to −0.3% (Column XIV). The comparison of *M_cr_* from the LTBeamN software with the results obtained from the Abaqus software (Column VIII) shows differences ranging from −3.3% to +1.7%.

The results in Table 7 show that the use of closed-section stiffeners (Figure 1c) in joints B and E of the H-grillage, compared with the use of flat stiffeners (Figure 1b), enhanced the elastic critical resistance of the critical beam B–E, and thus the ECR of the entire grillage. The increase in *M_cr_*, calculated according to Formula (9), ranged from +4% (for *L* = 7.5 m, *L*_1_ = 3 m) to over +21% (for *L* = 6 m, *L*_1_ = 12 m). Similar increases in the elastic critical resistance of the B–E critical beam were obtained for the triangular load, and for the beam loaded with a concentrated force.

**Figure 11:**
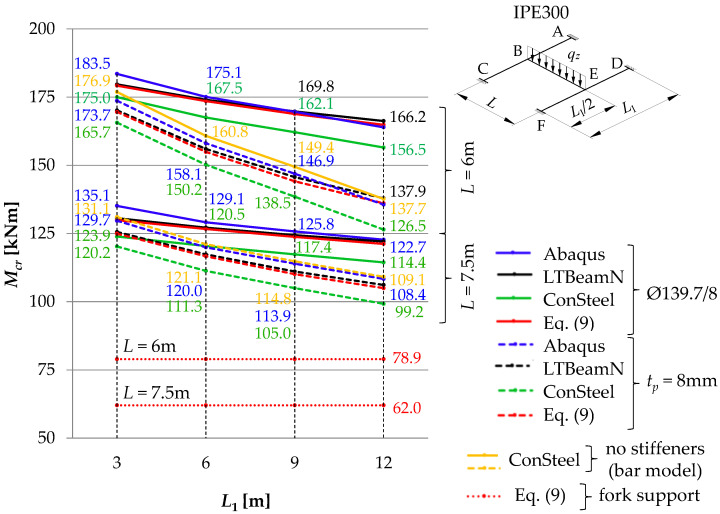
*M_cr_* of the critical (main) beam B–E with uniformly distributed load as a function of the span *L*_1_ of the supporting beams A–C and D–F.

**Figure 12:**
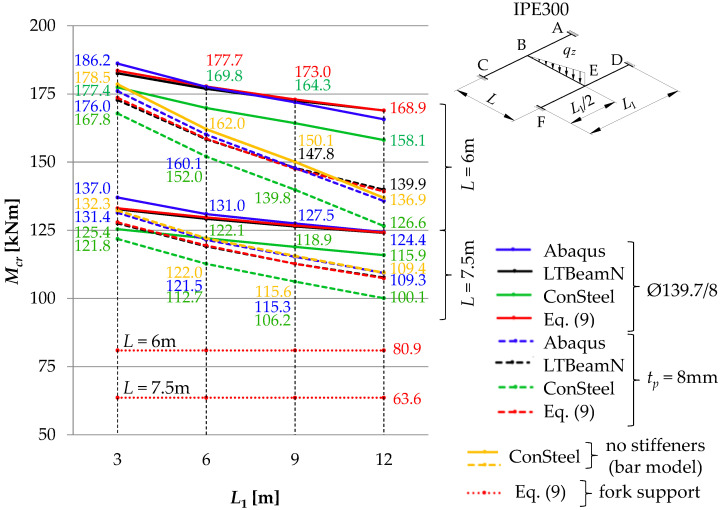
*M_cr_* of the critical (main) beam B–E with triangular load as a function of the span *L*_1_ of the supporting beams A–C and D–F.

**Figure 13:**
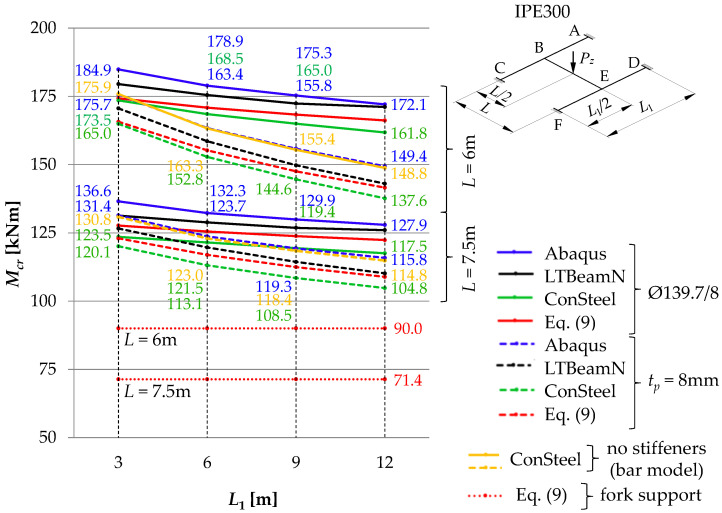
*M_cr_* of the critical (main) beam B–E with concentrated force as a function of the span *L*_1_ of the supporting beams A–C and D–F.

Figure 11, Figure 12 and Figure 13 show the pattern of variation in the critical moments of lateral torsional buckling of the B–E beam, determined according to Abaqus, ConSteel, LTBeamN and the approximation Formula (9) [11]. The critical beam was loaded at the level of the top flange (*z_g_* = +*h*/2) in the form of (a) uniformly distributed load (Figure 11), (b) triangularly distributed load (Figure 12), and (c) concentrated force at the midspan (Figure 13). The critical moments were determined, taking into account the closed-section stiffeners (continuous lines) or flat stiffeners (dashed lines) in the joints B and E. When the thin-walled bar model was used in ConSteel (orange lines), the stiffening at joints B and E of the grillage was not taken into account due to program limitation. For the critical beam with boundary conditions, as in the fork support, the critical moment obtained from Formula (9) is marked with horizontal dotted lines. In this case, the critical moment was estimated with the assumption of the fixity index *κ_ω_* = *κ_u_* = 0. The same *M_cr_* value was obtained from the FEM simulation in the LTBeamN software. The colours of the descriptions of the values of the critical moments of the B–E beams were associated with the colours of the lines showing the pattern of the *M_cr_* variation. Consequently, for a given span (*L* = 6 m or *L* = 7.5 m) of the main (critical) beam and the analysed type of stiffening (Ø139.7/8 or *t_p_* = 8 mm), the descriptions of the ordinates provide the maximum and minimum values of *M_cr_* for the given spans of the supporting beams *L*_1_. Additionally, all *M_cr_* values determined in the ConSteel software with the approximation of the grillage by the bar model are given.

The comparison of the diagrams (Figure 11, Figure 12 and Figure 13) shows that with an increase in the span *L*_1_ of the supporting beams (A–C and D–F), the critical moment of lateral torsion buckling of the critical (main) beam B–E decreases. At the same time, the effect of closed-section stiffeners on the stiffness of the B and E joints increases (continuous lines). The greatest efficiency in curbing the *M_cr_* drop caused by the use of closed-section stiffeners was obtained for the span *L* = 6 m of the main beam and *L*_1_ = 12 m of the supporting beams. Contribution of closed-section stiffeners increases with a decrease in the span of the B–E beam. The largest falls (Figure 11, Figure 12 and Figure 13) of the critical moment of lateral torsional buckling of the B–E beam, related to the increase in the span of the supporting beams, were obtained for flat stiffeners (dashed lines), especially for the span *L* = 6 m.

The *M_cr_* variation pattern determined on the basis of the values obtained from the ConSteel software, taking into account the bar model of the grillage (orange lines), are generally congruent with those obtained from the Abaqus software (dashed blue lines), in which the stiffness of the flat stiffeners at the joints B and E of the grillage was taken into account. That confirms the thesis about the negligible effect of classic flat stiffeners (in this case for *t_p_* = 8 mm) on the critical load of the grillage.

Taking into account the elastic restraint of the critical beam B–E at the joints resulted in a significant increase in the beam elastic critical resistance relative to the fork support. For a uniformly loaded beam (Figure 11) and a triangularly loaded beam (Figure 12), the increase in critical resistance ranged between +68% (at *L* = 7.5 m) and +127% (at *L* = 6 m). For a beam loaded with a concentrated force (Figure 13), the increase in *M_cr_* varied between +52% (at *L* = 7.5 m) and +93% (at *L* = 6 m). It should be emphasized that greater increases in the elastic critical resistance were obtained due to the use of closed-section stiffeners.

Table 8 shows a summary of the maximum percentage differences in the results (Method 1 vs. Method 3) obtained from the LTBeamN software, the M_LTB,EL,2_ algorithm, and from the Formula (9) [11] in relation to the Abaqus software. In the analysis, a total of 48 spatial models of the SBG H-type were performed in the Abaqus software, depending on (a) the span *L* and *L*_1_ of the component beams, (b) the type of stiffening of joints B and E (Figure 1b,c), and (c) the load diagram of the critical (main) beam B–E.

The critical moments of LTB (Table 8) of the H-grillage B–E critical beam, determined with the LTBeamN software and estimated using the M_LTB,EL,2_ algorithm and Formula (9), gave a very good approximation of the results obtained from numerical simulations in the Abaqus software (from −6.5% to +3.6%, Columns IV, V, and VII). When the *M_cr_* values obtained from the M_LTB,EL,2_ algorithm and Formula (9) with those produced through LTBeamN were compared, differences were found that ranged from −2.9% to +2.0% (Columns VI and VIII).

The application method of the approximation Formula (9) [11] and numerical simulations with the use of bar elements from the ConSteel software in the design of the H-grillage main (critical) beam is shown in the computational example (Appendix A).

## 8. Summary and Conclusions

In the study, the grillage ECR was determined. The ECR was measured by the external critical load (or *M_cr_*) from the condition of LTB of the weakest (critical) beam for the ideal (i.e., not showing up any imperfections) structural system model. Based on *M_cr_*, it is possible to determine the so-called relative slenderness of the critical beam and the relevant reduction factor for LTB taking into account the so-called integrated equivalent imperfection. Such an approach to deal with imperfections in actual steelwork components is accepted in Eurocode EN 1993-1-1 [1].

Based on the SBG elastic stability analysis, the following was found:

(1) When the interaction of beams in the H-grillage joints is taken into consideration in the computational model, it is possible to provide a more accurate representation of the actual performance of this structure class under load. Consequently, a more precise determination of the ECR is available, compared with estimation based on the fork support, which is most common in engineering design. It is obvious that when taking into account the effect of elastic restraint at joints, engineers must be cautious and check the actual stiffness of the joints.
(2) As the span *L*_1_ of the supporting beams increases, the *M_cr_* of the critical beam decreases. The greatest fall in *M_cr_*, related to the increase in the span of the supporting beams, was obtained for flat stiffeners (dashed lines in Figure 11, Figure 12 and Figure 13), especially for the main beam span *L* = 6 m. In this case, closed-section stiffeners should be used to increase *M_cr_*.
(3) The warping stiffness *α_ω_* of the welded grillage joints depends on their stiffening. The use of closed-section stiffeners considerably increases the degree of section fixity against warping. It also restricts the transmission of the bimoment between the joint component beams and enhances the grillage ECR. The use of classic flat stiffeners, however, practically does not increase *M_cr_*. In the analysed H-type grillage of concern, the use of closed-section stiffeners resulted in an increase in the ECR from 3.9% (*L* = 7.5 m, *L*_1_ = 3 m) to 15.2% (*L* = 6 m, *L*_1_ = 12 m) according to Abaqus (Table 6, Column VI), and also from 2.9% (*L* = 7.5 m, *L*_1_ = 3 m) to 17.6% (*L* = 6 m, *L*_1_ = 12 m) according to ConSteel—shell model (Table 6, Column VIII).
(4) The percentage increase in the ECR in the grillage with closed-section stiffeners (relative to grillage with flat stiffeners) is inversely proportional to the increase in the span *L*_1_ of supporting beams. Enhanced stiffness of the joints mitigates the decrease in *M_cr_* associated with a diminished stiffness of the supporting beams in the grillage plane. The greatest efficiency in the mitigation of the *M_cr_* drop due to the use of closed-section stiffeners was obtained for the span *L* = 6 m of the main beam and *L*_1_ = 12 m of the supporting beams (Figure 11, Figure 12 and Figure 13). The contribution of closed-section stiffeners increases with a decrease in the main beam span *L*.
(5) The means of determining the stiffness of the elastic action of the grillage joints reported in this paper, and the approximation Formula (9) proposed in [11], which at the same time takes into account the effect of elastic restraint against warping and against the lateral rotation of the grillage’s weakest (critical) beam, make it possible to produce a very good estimation of *M_cr_* compared with computations based on advanced FEM models (Abaqus, ConSteel). Determined in this way, *M_cr_* allows for an estimation of grillage ECR. Additionally, *M_cr_* can also be successfully applied to verify the FEM results.
(6) In the case of significant strength utilisation in the supporting (stiffening) beams relative to their critical buckling resistance (*M_cr,u_*), the reduction in the stiffness restraint of the critical beam can be conservatively estimated from Formulas (7) and (8). A more detailed approach to this issue will be the subject of further research and will be presented by the authors in another study.
(7) The results obtained from the FEM numerical simulations (Abaqus—solid model, ConSteel—shell model) show (Figure 11, Figure 12 and Figure 13) that the grillage ECR is conditioned by the lateral torsion buckling of the B–E critical beam. The critical resistance is nonlinearly dependent on the grillage geometry and the method of stiffening of welded intermediate joints B and E. The use of closed-section stiffeners resulted in ECR increasing from a few to over 21% compared with flat stiffeners (Table 6).
(8) From an engineering point of view, the comparison of results obtained with: Formula (9) [11], M_LTB,EL,2_ algorithm, and Abaqus (FEM) indicates their very good congruity (Table 7 and Table 8, Figure 11, Figure 12 and Figure 13). The LTB critical moments were obtained for (1) different variants of the coupling beam span (*L*) and supporting beams (*L*_1_), (2) different variants of nodal stiffening, and (3) different loading diagrams of the coupling beam.

It was demonstrated in the study that the congruence between the advanced FEM simulations for the whole structure and the analytical calculations for the designated critical beam make it possible for the results of both calculation methods to be verified against each other. The application of relatively simple approximation formulas allows a more advanced preliminary design. For basic structural systems, the formulas can also be employed in the standard design. Due to such an approach, the safety of structures can already be improved at the design stage.

It seems necessary to conduct further research into the stability and design resistance of beam grillages with the use of the analytical computational model of the critical beam elastically restrained in adjacent (stiffening) beams. This method of analysis will be continued by the authors.

The explicit account of geometric and structural imperfections (including welding stress) in the grillage component beams makes it possible to determine the non-linear load–displacement path and limit strength. This approach, however, requires further advanced analyses. They should include, among others, the determination of the mutual effect of the shape imperfections in component beams of H-type grillages while taking into account nodal properties. This would especially concern nodes with closed-section stiffeners. It seems necessary to carry out experimental investigations into SGB in order to validate non-linear FEM models. Obtaining failure and limit strength mechanisms in the experimental way would allow for a better assessment of the structural system capacity in the elastic–plastic range, taking into account imperfections.

A natural trend in design methods is to take into consideration real design solutions of joints, which generate complex conditions for the support of grillage component beams. Research in this area primarily aims to assess the safety of beam grillage structures, while taking into account the potential resistance reserves that result from the solutions applied at the joints.

## Figures and Tables

**Table 1:** The ECR (*q_z,cr_*) of H-type grillage from the condition of lateral torsional buckling of the main (critical) beam B–E, uniformly loaded (*z_g_* = +*h*/2), from Abaqus (solid model).

Item	*L* [m]	*L*_1_ [m]	*z_g_* [mm]	*q_z,cr_* [kN/m] Acc. Abaqus		% [-] VI-V
				Stiffener *t_p_* = 8 mm	Stiffener Ø139.7/8	
I	II	III	IV	V	VI	VII
1	6	3	150	38.6	40.8	5.7
2		6		35.1	38.9	10.8
3		9		32.6	37.7	15.6
4		12		30.1	36.4	20.9
5	7.5	3		18.4	19.2	4.3
6		6		17.1	18.4	7.6
7		9		16.2	17.9	10.5
8		12		15.4	17.4	13.0

**Table 2:** The ECR (*q_z,cr_*) of H-type grillage from the condition of lateral torsional buckling of the main (critical) beam B–E, uniformly loaded (*z_g_* = +*h*/2), from ConSteel (bar model).

Item	*L* [m]	*L*_1_ [m]	*z_g_* [mm]	*q_z,cr_* [kN/m] Acc. ConSteel —Bar Model
				No Stiffener
I	II	III	IV	V
1	6	3	150	39.31
2		6		35.73
3		9		33.21
4		12		30.59
5	7.5	3		18.64
6		6		17.22
7		9		16.33
8		12		15.52

**Table 3:** The ECR (*q_z,cr_*) of H-type grillage from the condition of lateral torsional buckling of the main (critical) beam B–E, uniformly loaded (*z_g_* = +*h*/2), from ConSteel (shell model).

Item	*L* [m]	*L*_1_ [m]	*z_g_* [mm]	*q_z,cr_* [kN/m] Acc. ConSteel - Shell Model		% [-] VI-V
				Stiffener *t_p_* = 8 mm	Stiffener Ø139.7/8	
I	II	III	IV	V	VI	VII
1	6	3	150	36.81	38.89	5.7
2		6		33.38	37.23	11.5
3		9		30.78	36.03	17.1
4		12		28.10	34.78	23.8
5	7.5	3		17.10	17.62	3.0
6		6		15.83	17.13	8.2
7		9		14.93	16.69	11.8
8		12		14.11	16.27	15.3

**Table 4:** Critical moment (*M_cr_*) of the critical beam B–E of H-shaped grillage, uniformly loaded (*z_g_* = +*h*/2), from analytical methods.

Item	*L* [m]	*L*_1_ [m]	*z_g_* [mm]	*M_cr_* [kNm] Acc. Analytical Method				% [-] VIII-VI
				Stiffener *t_p_* = 8 mm		Stiffener Ø139.7/8		
				M_LTB,EL,2_	Equation (9) [11]	M_LTB,EL,2_	Equation (9) [11]	
I	II	III	IV	V	VI	VII	VIII	IX
1	6	3	150	170.5	169.6	180.2	179.2	5.7
2		6		156.3	154.9	174.5	173.5	12.0
3		9		145.9	144.2	170.1	168.8	17.1
4		12		138.0	136.1	166.5	164.9	21.2
5	7.5	3		125.9	124.9	131.0	129.9	4.0
6		6		117.6	116.6	127.5	126.6	8.6
7		9		111.3	110.1	124.7	123.8	12.4
8		12		106.3	105.0	122.3	121.2	15.4

**Table 5:** Critical moment (*M_cr_*) of the critical beam B–E of H-shaped grillage, uniformly loaded (*z_g_* = +*h*/2), from LTBeamN (thin-walled bar model).

Item	*L* [m]	*L*_1_ [m]	*z_g_* [mm]	*M_cr_* [kNm] Acc. Bar Model		% [-] VI-V
				Stiffener *t_p_* = 8 mm	Stiffener Ø139.7/8	
I	II	III	IV	V	VI	VII
1	6	3	150	170.1	179.7	5.6
2		6		156.0	174.1	11.6
3		9		145.7	169.8	16.5
4		12		137.9	166.2	20.5
5	7.5	3		125.6	130.6	4.0
6		6		117.4	127.2	8.3
7		9		111.1	124.4	12.0
8		12		106.2	122.1	15.0

**Table 6:** Comparison of *P_z,cr_* for the H-type grillage loaded with the concentrated force (*z_g_* = +*h*/2) on the B–E main (critical) beam.

Item	Stiffener	*L* [m]	*L*_1_ [m]	*z_g_* [mm]	*P_z,cr_* [kN]			% [-]		
					Method 1	Method 2		VII-VI	VIII-VI	VIII-VII
					Abaqus– Solid Model	ConSteel– Bar Model	ConSteel— Shell Model			
I	II	III	IV	V	VI	VII	VIII	IX	X	XI
1	*t_p_* = 8 mm	6	3	150	117.11	117.25	109.97	0.1	−6.1	−6.2
2			6		108.93	108.84	101.88	−0.1	−6.5	−6.4
3			9		103.89	103.60	96.41	−0.3	−7.2	−6.9
4			12		99.61	99.19	91.76	−0.4	−7.9	−7.5
5		7.5	3		70.09	69.78	64.05	−0.4	−8.6	−8.2
6			6		65.98	65.59	60.32	−0.6	−8.6	−8.0
7			9		63.64	63.16	57.84	−0.8	−9.1	−8.4
8			12		61.78	61.25	55.88	−0.9	−9.6	−8.8
9	Ø139.7/8	6	3	150	123.25	117.25	115.69	−4.9	−6.1	−1.3
10			6		119.29	108.84	112.35	−8.8	−5.8	3.2
11			9		116.89	103.60	110.00	−11.4	−5.9	6.2
12			12		114.73	99.19	107.87	−13.5	−6.0	8.8
13		7.5	3		72.83	69.78	65.89	−4.2	−9.5	−5.6
14			6		70.53	65.59	64.79	−7.0	−8.1	−1.2
15			9		69.29	63.16	63.68	−8.8	−8.1	0.8
16			12		68.23	61.25	62.69	−10.2	−8.1	2.4

**Table 7:** Comparison of *M_cr_* for uniformly loaded (*z_g_* = +*h*/2) B–E critical beam of the H-grillage.

Item	Stiffener	*L* [m]	*L*_1_ [m]	*z_g_* [mm]	*M_cr_* [kNm]		% [-]	*M_cr_* [kNm]	% [-]		*M_cr_* [kNm]	% [-]	
					Abaqus	LTBeamN	VII-VI	M_LTB,EL,2_	IX-VI	IX-VII	Equation (9) [11]	XII-VI	XII-VII
I	II	III	IV	V	VI	VII	VIII	IX	X	XI	XII	XIII	XIV
1	*t_p_* *	6	3	150	173.7	170.1	−2.1	170.5	−1.8	0.2	169.6	−2.4	−0.3
2			6		158.1	156.0	−1.3	156.3	−1.1	0.2	154.9	−2.0	−0.7
3			9		146.9	145.7	−0.8	145.9	−0.7	0.1	144.2	−1.8	−1.0
4			12		135.6	137.9	1.7	138.0	1.8	0.1	136.1	0.4	−1.3
5		7.5	3		129.7	125.6	−3.2	125.9	−2.9	0.2	124.9	−3.7	−0.6
6			6		120.0	117.4	−2.2	117.6	−2.0	0.2	116.6	−2.8	−0.7
7			9		113.9	111.1	−2.5	111.3	−2.3	0.2	110.1	−3.3	−0.9
8			12		108.4	106.2	−2.0	106.3	−1.9	0.1	105.0	−3.1	−1.1
9	Ø **	6	3	150	183.5	179.7	−2.1	180.2	−1.8	0.3	179.2	−2.3	−0.3
10			6		175.1	174.1	−0.6	174.5	−0.3	0.2	173.5	−0.9	−0.3
11			9		169.6	169.8	0.1	170.1	0.3	0.2	168.8	−0.5	−0.6
12			12		163.9	166.2	1.4	166.5	1.6	0.2	164.9	0.6	−0.8
13		7.5	3		135.1	130.6	−3.3	131.0	−3.0	0.3	129.9	−3.8	−0.5
14			6		129.1	127.2	−1.5	127.5	−1.2	0.2	126.6	−1.9	−0.5
15			9		125.8	124.4	−1.1	124.7	−0.9	0.2	123.8	−1.6	−0.5
16			12		122.7	122.1	−0.5	122.3	−0.3	0.2	121.2	−1.2	−0.7

* Flat stiffener (*t_p_* = 8 mm), ** closed-section stiffener (Ø139.7/8).

**Table 8:** Summary of the percentage differences of *M_cr_* for the B–E critical beam of the H-grillage.

Loading Diagram of the B–E Beam	*z_g_* [mm]	Stiffener	LTBeamN vs. Abaqus [%]	M_LTB,EL,2_ vs. Abaqus [%]	M_LTB,EL,2_ vs. LTBeamN [%]	Equation (9) [11] vs. Abaqus [%]	Equation (9) [11] vs. LTBeamN [%]
I	II	III	IV	V	VI	VII	VIII
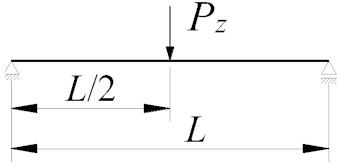	150	*t_p_* = 8 mm	−4.8 ÷ −2.9	−3.4 ÷ −1.1	1.4 ÷ 1.9	−6.4 ÷ −5.0	−2.9 ÷ −1.0
		Ø139.7/8	−3.9 ÷ −0.6	−1.9 ÷ 0.3	0.9 ÷ 2.0	−6.5 ÷ −3.4	−2.9 ÷ −2.4
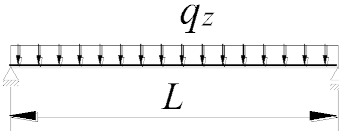	150	*t_p_* = 8 mm	−3.2 ÷ 1.7	−2.9 ÷ 1.8	0.1 ÷ 0.3	−3.6 ÷ 0.3	−1.3 ÷ −0.3
		Ø139.7/8	−3.3 ÷ 1.4	−3.0 ÷ 1.6	0.2 ÷ 0.3	−3.9 ÷ 0.6	−0.8 ÷ −0.3
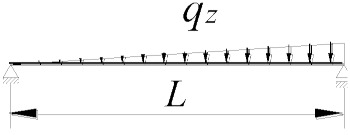	150	*t_p_* = 8 mm	−3.0 ÷ 3.1	−2.3 ÷ 3.6	0.6 ÷ 0.7	−2.7 ÷ 2.6	−0.5 ÷ 0.5
		Ø139.7/8	−3.1 ÷ 2.0	−2.5 ÷ 2.5	0.5 ÷ 0.7	−2.9 ÷ 1.9	0.1 ÷ 0.5

## Data Availability

The data presented in this study are available on request from the corresponding author.

## Appendix A

*Problem.* For the static diagram of SBG H-type (Figure 1a) loaded with a concentrated force *P_z_* = 70 kN, check the design condition of the lateral buckling resistance of the main (coupling) beam according to EN 1993-1-1 [1] for two variants of stiffening of joints B and E: (a) variant 1—flat stiffener *t_p_* = 8 mm, (b) variant 2—closed-section stiffener Ø139.7/8. The comparative calculations took into account the degree of reduction in the stiffness of the support beams as a result of their loading with reactions from the main beam (results marked with *).

*Data: L* = 6 m, *L*_1_ = 9 m, beam section: IPE300 (*W_pl,y_* = 629.86 cm^3^), steel: S355 (*f_y_* = 355 MPa), class 1 section in bending: *M_Ed_* = 105 kNm.

*Solution.*

Simulation results in the ConSteel software—the first critical force *P_z,cr,_*_1_ = 103.6kN (lateral torsional buckling of the B–E critical beam according to one half-wavelength), the fourth critical force *P_z,cr,_*_4_ = 1027.2 kN (lateral torsional buckling of the supporting beams).

Stiffness coefficients of the elastic restraint of the main (critical) beam in joints B and E:

(a) Variant 1—acc. Formulas (3), (4): *α_ω_* = 47.00 kNm^3^/rad, *α_u_* = 2254.93 kNm

acc. Formulas (7), (8): *α_ω_** = 42.30 kNm^3^/rad, *α_u_** = 2029.44 kNm

(b) Variant 2—acc. Formulas (4), (5), (6): *α_ω_* = 384.41 kNm^3^/rad, *α_u_* = 2254.93 kNm

acc. Formulas (7), (8): *α_ω_** = 345.97 kNm^3^/rad, *α_u_** = 2029.44 kNm

Fixity index according to Formulas (1), (2):

(a) Variant 1: *κ_ω_* = 0.842, *κ_u_* = 0.842, *κ_ω_** = 0.828, *κ_u_** = 0.828
(b) Variant 2: *κ_ω_* = 0.978, *κ_u_* = 0.842, *κ_ω_** = 0.975, *κ_u_** = 0.828

Critical moment for lateral torsional buckling of the B–E critical beam according to Formula (9) [11]:

(a) Variant 1: *M_cr_*^(1)^ = 147.54 kNm, *M_cr_*^(1)*^ = 145.47 kNm (−1.4%)
(b) Variant 2: *M_cr_*^(2)^ = 168.41 kNm, *M_cr_*^(2)*^ = 167.07 kNm (−0.8%)

Reduction factor for lateral torsional buckling (general case) [1]:

(a) Variant 1: *λ_LT_*^(1)^ = 1.158, *α_LT_*^(1)^ = 0.21, *Ф_LT_*^(1)^ = 1.271, *χ_LT_*^(1)^ = 0.557

*λ_LT_*^(1)*^ = 1.166, *α_LT_*^(1)^ = 0.21, *Ф_LT_*^(1)*^ = 1.281, *χ_LT_*^(1)*^ = 0.552 (−0.9%)

(b) Variant 2: *λ_LT_*^(2)^ = 1.084, *α_LT_*^(2)^ = 0.21, *Ф_LT_*^(2)^ = 1.180, *χ_LT_*^(2)^ = 0.607

*λ_LT_*^(2)*^ = 1.088, *α_LT_*^(2)^ = 0.21, *Ф_LT_*^(2)*^ = 1.185, *χ_LT_*^(2)*^ = 0.604 (−0.5%)

Lateral torsional buckling resistance moment [1]:

(a) Variant 1: *M_b,Rd_*^(1)^ = 124.55 kNm, *M_b,Rd_*^(1)*^ = 123.43 kNm (−0.9%)
(b) Variant 2: *M_b,Rd_*^(2)^ = 135.73 kNm, *M_b,Rd_*^(2)*^ = 135.05 kNm (−0.5%)

Bearing capacity condition [1]:

(a) Variant 1: *M_Ed_*/*M_b,Rd_*^(1)^ = 0.843 < 1.0 (load capacity fulfilled)

*M_Ed_*/*M_b,Rd_*^(1)*^ = 0.851 < 1.0

(b) Variant 2: *M_Ed_*/*M_b,Rd_*^(2)^ = 0.774 < 1.0 (load capacity fulfilled)

*M_Ed_*/*M_b,Rd_*^(2)*^ = 0.777 < 1.0

Checking the load capacity condition of the B–E main beam with fork support:

*M_cr_*^(3)^ = 90.03 kNm, *χ_LT_*^(3)^ = 0.380, *M_b,Rd_*^(3)^ = 84.97 kNm

*M_Ed_*/*M_b,Rd_*^(3)^ = 1.236 > 1.0 (load capacity condition is not met)

*Conclusions from the example:*

(1) Taking into account the conditions of elastic restraint of the main (coupling) beam in the supporting beams allowed for a more economical assessment of its design lateral buckling resistance and fulfilment of the load capacity condition according to EN 1993-1-1 [1].
(2) The use of closed-section stiffeners with at joints B and E of the H-grillage, compared to the use of flat stiffeners, resulted in an increase in *M_cr_* by 14% and an increase in the design load capacity *M_b,Rd_* by 9%.
(3) Analytical consideration of the spatial interaction of the grillage beams made it possible to maintain the resistance condition of the main (coupling) beam B–E without the need to increase its cross-section.
(4) Due to the significant difference between *M_cr_* for the critical (main) beam in relation to *M_cr,u_* for the supporting (stiffening) beams, the reduction in the design resistance was only −0.9% (variant 1) and −0.5% (variant 2). In the case of greater strength utilisation of the supporting beams, the degree of reduction of joint stiffness can be conservatively determined from Formulas (7) and (8).
(5) In computations, the use of the fork support model for the main (coupling) beam design resulted in a conservative assessment of the beam resistance (*M_b,Rd_*^(3)^). Due to the simple analytical model, such a simplification is often found in engineering practice. This is a safe estimate, giving a lower determination of the lateral buckling resistance of the beam. However, as already mentioned, due to the spatial interaction of the component beams and the stiffeners of the joints of H-grillage, especially closed-section stiffeners, use of the fork support model for the weakest (critical) beam is not an economical solution. In the computational example, adopting such a method of supporting the main beam resulted in failure to meet the design condition according to EN 1993-1-1 [1]. In order to satisfy the condition above, it would be necessary to increase the beam cross-section to at least IPE360.

## References

[1] (2005). Eurocode 3: Design of Steel Structures—Part 1-1: General Rules and Rules for Buildings.

[2] (2005). Eurocode 3: Design of Steel Structures—Part 1-8: Design of joints.

[3] Koczubiej S., Cichoń C. (2014). Global static and stability analysis of thin-walled structures with open cross-section using FE shell-beam models. Thin-Walled Struct..

[4] Shayan S., Rasmussen K. (2014). A model for warping transmission through joints of steel frames. Thin-Walled Struct..

[5] Kujawa M. (2009). Static and sensitivity analysis of grids made of thin-walled members. Theoretical Analysis and Experimental Research.

[6] Szymczak C., Kreja I., Mikulski T., Kujawa M. (2003). Sensitivity Analysis of Beams and Frames Made of Thin-Walled Members.

[7] Vaszilievits-Sömjén B., Szalai J. Simple Superelement Model of Warping Transfer in Moment Connections Between I Sections. Proceedings of the Ninth International Conference on Advances in Steel Structures.

[8] Vaszilievits-Sömjén B., Szalai J., Rad M.M. (2019). Validation of simple superelement model of warping transfer in moment connections of portal frames. Ce/Pap. Online Collect. Conf. Pap. Civ. Eng..

[9] Szychowski A. (2016). Computation of Thin-Walled Cross-Section Resistance to Local Buckling with the Use of the Critical Plate Method. Arch. Civ. Eng..

[10] Giżejowski M. (2001). Lateral buckling of steel beams with limited rotation ability at supports. Inżynieria I Bud..

[11] Piotrowski R., Szychowski A. (2019). Lateral Torsional Buckling of Steel Beams Elastically Restrained at the Support Nodes. Appl. Sci..

[12] Amara S., Kerdal D.E., Jaspart J.P. (2008). Effect of end connection restraints on the stability of steel beams in bending. Adv. Steel Constr..

[13] Piotrowski R., Szychowski A. (2022). The Effect of Steel Beam Elastic Restraint on the Critical Moment of Lateral Torsional Buckling. Materials.

[14] Lee H.E., Nguyen C.T., Moon J.H., Joo H.S. (2011). Lateral-torsional buckling of discretely-braced i-girder. Procedia Eng..

[15] Nguyen C.T., Moon J., Le V.N., Lee H.E. (2010). Lateral–torsional buckling of I-girders with discrete torsional bracings. J. Constr. Steel Res..

[16] Valentino J., Trahair N.S. (1998). Torsional Restraint against Elastic Lateral Buckling. J. Struct. Eng..

[17] Stroetmann R. Lateral torsional and distortional buckling of cross-connected beams. Proceedings of the Eighth International Conference on Advances in Steel Structures.

[18] Piotrowski R., Siedlecka M. (2020). Point Protection of Primary Beams of Steel Grillages Against Lateral Torsional Buckling. Adv. Sci. Technol. Res. J..

[19] Yura J. (2001). Fundamentals of beam bracing. Eng. J..

[20] Gosowski B. (2007). Non-uniform torsion of stiffened open thin-walled members of steel structures. J. Constr. Steel Res..

[21] Gosowski B., Redecki M. (2012). Lateral-torsional buckling moments for I-beams with local lateral restraints. Inżynieria I Bud..

[22] Park Y.-M., Hwang S.-Y., Hwang M.-O., Choi B.H. (2010). Inelastic buckling of torsionally braced I-girders under uniform bending: I. Numerical parametric studies. J. Constr. Steel Res..

[23] Gull J.H., Azizinamini A., Helwig T.A. (2017). Comparison of detailing methods for straight skewed steel I-girder bridges. J. Constr. Steel Res..

[24] Yura J., Helwig T., Herman R., Zhou C. (2008). Global Lateral Buckling of I-Shaped Girder Systems. J. Struct. Eng..

[25] Zhao Q., Yu B., Burdette E.G. (2010). Effects of Cross-Frame on Stability of Double I-Girder System under Erection. Transp. Res. Board.

[26] Adamakos T., Vayas I., Petridis S., Iliopoulos A. (2011). Modeling of curved composite I-girder bridges using spatial systems of beam elements. J. Constr. Steel Res..

[27] Gocál J., Odrobiňák J. (2020). On the Influence of Corrosion on the Load-Carrying Capacity of Old Riveted Bridges. Materials.

[28] Rageh A., Sun C., Linzell D.G., Puckett J.A. (2022). Dataset for large-scale, lateral-torsional buckling tests of continuous beams in a grillage system. Data Brief.

[29] Sun C., Linzell D.G., Puckett J.A., Akintunde E., Rageh A. (2022). Experimental Study of Continuous-Beam Lateral Torsional-Buckling Resistance with a Noncomposite Concrete Deck. J. Struct. Eng..

[30] Kong X., Zhou H., Zheng C., Pei Z., Yuan T., Wu W. (2021). Research on the dynamic buckling of a typical deck grillage structure subjected to in-plane impact Load. Mar. Struct..

[31] Piotrowski R., Szychowski A. (2018). Impact of support closed section ribs on the critical moment for lateral torsional buckling of steel beams. Struct. Environ..

[32] Pi Y.-L., Trahair N.S. (2000). Distortion and warping at beam supports. J. Struct. Eng..

[33] Piotrowski R., Szychowski A. Critical resistance of a beam grillage in FEM simulations and analytical calculations. Proceedings of the International Conference Selected Issues in Building Structures Design.

[34] (2012). Abaqus 6.12. Abaqus/CAE User’s Manual.

[35] ConSteel User Manuals. https://consteelsoftware.com/downloads/.

[36] Vlasov V.Z. (1961). Thin-Walled Elastic Beams.

[37] Cross H. (1932). Analysis of Continuous Frames By Distributing Fixed-End Moments. Am. Soc. Civ. Eng..

[38] Galéa Y. (2003). Moment critique de déversement élastique de poutres fléchies. Présentation du logiciel LTBEAM. Rev. Constr. Métallique. CTICM.

